# Variation of the Bacterial Community in the Rhizoplane Iron Plaque of the Wetland Plant *Typha latifolia*

**DOI:** 10.3390/ijerph15122610

**Published:** 2018-11-22

**Authors:** Haochun Chi, Lu Yang, Wenjing Yang, Yuanyuan Li, Ziwu Chen, Lige Huang, Yuanqing Chao, Rongliang Qiu, Shizhong Wang

**Affiliations:** 1 School of Environmental Science and Engineering, Sun Yat-sen University, Guangzhou 510275, China; chihch@mail2.sysu.edu.cn (H.C.); yanglu26@mail2.sysu.edu.cn (L.Y.); yangwj26@mail2.sysu.edu.cn (W.Y.); liyy97@mail2.sysu.edu.cn (Y.L.); chenzw29@mail2.sysu.edu.cn (Z.C.); huanglig@mail2.sysu.edu.cn (L.H.); chaoyuanq@mail.sysu.edu.cn (Y.C.); eesqrl@mail.sysu.edu.cn (R.Q.); 2 Guangdong Provincial Key Lab of Environmental Pollution Control and Remediation Technology, Guangzhou 510275, China; 3 Guangdong Provincial Engineering Research Center for Heavy Metal Contaminated Soil Remediation, Guangzhou 510275, China

**Keywords:** wetland, mine tailing, iron plaque, *Typha latifolia*, bacterial community

## Abstract

The survival of wetland plants in iron, sulfur and heavy metals-rich mine tailing ponds has been commonly attributed to the iron plaque (IP) on the root surface that acts as a protective barrier. However, the contribution of bacteria potentially regulates the iron-sulfur cycle and heavy metal exclusion at the root surface has not been studied in depth, particularly from a microbial ecology perspective. In this study, a pot experiment using *Typha latifolia*, a typical wetland plant, in non-polluted soil (NP) and tailing soil (T) was conducted. Samples from four zones, comprising non-rhizosphere soil (NR), rhizosphere soil (R) and internal (I) and external (E) layers of iron plaque, were collected from the NP and T and analyzed by 16S rRNA sequencing. Simpson index of the genus level showed greater diversities of bacterial community in the NP and its I zone is the most important part of the rhizosphere. PICRUSt predicted that the I zones in both NP and T harbored most of the functional genes. Specifically, functional genes related to sulfur relay and metabolism occurred more in the I zone in the T, whereas those related to iron acquisition and carbon and nitrogen circulation occurred more in the I zone in the NP. Analysis of dominant bacterial communities at genus level showed highest abundance of heavy metal resistant genus *Burkholderia* in the E zones in both soils, indicating that heavy metal resistance of *Typha latifolia* driven by *Burkholderia* mainly occurred at the external layer of IP. Moreover, many bacterial genera, such as *Acidithiobacillus*, *Ferritrophicum*, *Thiomonas*, *Metallibacterium* and *Sideroxydans,* involved in iron and sulfur metabolisms were found in the T and most showed higher abundance in the I zone than in the other zones. This work, as the first endeavor to separate the iron plaque into external and internal layers and investigate the variations of the bacterial communities therein, can provide an insight for further understanding the survival strategy of wetland plants, e.g., *Typha latifolia*, in extreme environment.

## 1. Introduction

The past several decades have seen a revolution in characterizing the bacterial communities that perform important roles in wetlands. Significant progress has been made in developing modern analyses and gene technologies based on 16S rDNA gene sequencing. For example, clone gene library technology has been used to identify the rhizobacterial communities of plants in the constructed wetland of Cuihu Lake in Beijing [1,2]. Wetland rhizobacterial communities play an important role in the transformation of nitrogen, phosphorus, and sulfur and in the degradation of many types of organic composites, which is important to water and sediment clean up in wetlands [1]. In the rhizobacterial communities of the wetland plants *Typha latifolia* and *Phragmites australis*, some groups of bacteria that fix nitrogen and phosphorus and degrade organic carbon occur, and include many types of bacteria that can potentially degrade polyamines, cyanide, urea, and other compounds [2]. In wetlands located adjacent to or near the outlets of mine tailing ponds, rhizobacteria closely related to the regulation of iron, sulfur and heavy metal speciation warrant attention for exploring on-site control of heavy metal pollution.

Previous studies of microbial community structure of wetland plants show that the root is the most active area of microorganisms. The various surface structures of roots, rhizomes and rhizoids stimulate aerobic and anaerobic mineralization processes, and lead to concentration gradients of root exudates and gases such as N_2_, O_2_, and CO_2_. Therefore, a diversified microorganism community structure is formed around these structures. Moreover, the oxygen released from the plants, residue accumulation and rhizosphere effects also contribute to the diversity of wetland microorganisms in soil. In an integrated vertical-flow constructed wetland composed of eutrophic landscape water, the highest diversity of the bacterial community was observed in the surface of the wetland soil [3], which was the result of the regulatory effects of the wetland plant roots.

Having different main plant and sediment constituents, different wetland systems have varying amounts and types of rhizosphere microorganisms and, hence, different water and sediment pollutant removal efficiencies. In addition, the formation of iron plaque (IP) at the root surface, favored by the radial oxygen loss (ROL) exclusively found in wetland plants, influences pollutant-removal efficiencies. To date, much attention has been given to the spatial distributions of heavy metals and the microorganisms in the iron plaque of wetland plants in an effort to identify the microbial contribution to heavy metal sequestration at the root surface. Hansel et al. determined the metal distributions on and within the roots of the wetland plant *Phalaris arundinacea* by fluorescence microtomography, and found they were strongly associated with the microorganisms therein [4]. They also found some reductive anaerobic microorganisms in the micro-oxic environment at the root surface, which was unexpected. Similarly, Stubner et al. found that thiosulfate-oxidizing and sulfate-reducing bacteria were abundant in the bulk (non-rhizosphere) and rhizosphere of paddy soil [5]. They also identified a closed sulfur cycle in which reduced sulfur compounds were reoxidized by sulfur-oxidizing bacteria in the rhizosphere, allowing the sulfate produced to be consumed by sulfate-reducing bacteria.

Typically, microbial oxidation and reduction occur strictly under aerobic and anaerobic conditions, respectively. Submerged wetland sediments and long-term flooded paddy fields are generally believed to be oxygen-free; the ROL of plants in these environments alters this condition by introducing a sharp oxygen gradient from the root surface to the bulk soil. Thus, studies on the spatial distributions of the oxidative and reductive microorganisms along this gradient under varied oxygen supply conditions and at different sites can provide insight into the microbial contributions to different element cycles. Previously, iron-oxidizing bacteria were proven to be related to the formation of iron plaque on wetland plant roots [6], which can be attributed to the ROL. However, the coexistence of oxidative (aerobic) and reductive (anaerobic) microorganisms related to the iron and sulfur cycles has also been observed in the oxidized rhizosphere of wetland plants. Moreover, the amount of anaerobic sulfate reducing rhizobacteria on the root surface has been found to be significantly higher than that in the non-rhizosphere soil of wetland plants [7], which suggests that the oxygen gradient resulting from the ROL is not the sole factor modulating the microbial distribution.

In an extreme environment, with limited nutrient elements and excessive acidity and heavy metals, e.g., a mine tailing pond, both microorganisms and plants have strategies to cope with the associated stresses. At the microscale, microbial extracellular polymers in the iron plaque can help microorganisms resist the stresses of extremely acidic and toxic environments [8]. The functional microorganisms existing in the rhizosphere environment, i.e., on the root surface and in the iron plaque, can promote plant growth by facilitating nitrogen and phosphorus cycling, e.g., the nitrification of ammonia and dissolution of phosphate, and thereby increasing their availability to plants [9]. Wetland plants *Typha latifolia* and *Phragmites australis* are commonly dominant plant species in mine tailing pond wetlands. Many common bacterial genera have the ability to help *Typha latifolia* resist heavy metal stress by absorbing Cd, Cu, Pb, Zn and other heavy metals [10,11]. This ability confers *Typha latifolia* strong resistance to stress conditions, and this plant has been widely used for heavy metal pollution control and remediation in tailing ponds. Previous research on *Typha latifolia* mainly focused on its absorption and accumulation of heavy metals [12], with the mechanism of heavy metal absorption and transport in iron plaque being a research hotspot. However, few studies have focused on the microbial community in the iron plaque on the root surface. Thus, the structure of the microbial community and its contribution to matter cycling in iron plaque, the rhizosphere and the non-rhizosphere remain unknown.

It may be expected that the iron plaque and rhizosphere and non-rhizosphere soil harbor different microbial community structures. The root ROL leads to an oxygen gradient over different regions around the root, which might favor particular microorganisms with some specific properties [13,14]. However, this possibility has yet to be confirmed. This study, based on previous research, focused on the internal aerobic layer and external anaerobic layer of iron plaque in the *Typha latifolia* rhizosphere. We compared the differences in bacterial abundances between the internal and external layers of iron plaque by a molecular biotechnique and bioinformatics method and investigated the impacts of some iron and sulfur cycle-related bacteria in rhizosphere soil (R), non-rhizosphere soil (NR), and the internal (I) and external (E) layers of iron plaque in non-polluted soil (NP) and tailing soil (T) planted with *Typha latifolia*.

## 2. Materials and Methods

### 2.1. Tested Soil and Typha latifolia

Tailing soil (T) for the indoor experiment was obtained from a mine tailing pond that had *Typha latifolia* as the sole colonizing plant species on Dabao Mountain, Guangdong Province, China (24°32′31″ N, 113°43′20″ E). To guarantee the soil was representative of the source soil, three 1 m^2^ quadrats were established in an area of the pond without *Typha latifolia* colonization, and soil was randomly collected from these quadrats at 0–30 cm depth. After an adequate amount of soil was obtained to serve as one of the experimental soils, the soils from the three quadrats were evenly mixed. The non-polluted soil (NP) was obtained from an experimental farmland of South China Agricultural University in Guangzhou, China (23°7′12″ N 113°15′00″ E), air-dried for one week and sieved through a 20-mesh sieve. The physical and chemical properties of the T and NP are shown in Table 1. The total concentrations of C, N, and S were determined by EA (Flash 2000 HT, Thermo Scientific, Waltham, MA, USA), and the concentrations of heavy metals were determined by ICP-OES (Optima 5300DV, Perkin Elmer, Waltham, MA, USA). The concentrations of nitrogen in the T and of sulfur in the NP were too low to be detected by the EA. The seedlings of *Typha latifolia* were provided by Jinfoshan Ecological Farm in Jiangsu Province, China.

### 2.2. Experimental Design

To allow *Typha latifolia* to grow under flooded condition (mimicking wetland conditions), the tailing soil (T) or non-polluted soil (NP) was placed in watertight pots at 1 kg per pot, and deionized water was added to each pot at a water to soil ratio of 1.25:1 (*v*/*v*). All the pots were maintained at room temperature for 3 days prior to the transplantation of *Typha latifolia*. The seedlings of *Typha latifolia* in uniform size of 15 cm height and with 4 leaves were transplanted in the pots, with two seedlings per pot. Eight replicates were prepared for each T and NP treatment. All the pots with *Typha latifolia* seedlings were placed in a greenhouse with natural light and dark condition, temperature was controlled at 25 °C/20 °C for day/night, humidity was maintained at 80%. The water coverage in each pot was maintained at a depth of 1.5 cm throughout the experiment. Two months after the transplantation of *Typha latifolia* seedings, sample collection was conducted.

### 2.3. Sample Preparation

Soil samples were collected from either bulk soil (non-rhizosphere, NR) or rhizosphere soil (R). In addition, iron plaques were sampled, and each plaque sample was separated into external (E) and internal (I) layers. Samples were collected according to literatures [6,15] with modification. Bulk soil samples were collected from areas 10 cm distant from the root, without *Typha latifolia* root colonization and served as non-rhizosphere soil (NR). A sequenced washing method using deionized water, PBS-S solution and hydroxylamine hydrochloride solution, respectively, for the collection of rhizosphere soil (R), external layer (E) of iron plaque and internal layer (I) of iron plaque extraction was employed as following. Roots with a total length of 20 cm were collected, manually shaken to remove the surface soil, and quickly washed three times in sterile deionized water. No agitation was included in this process. The soil washed from the roots along with the washing water were collected in a centrifuge tube and centrifuged at 16,000× *g* for 4 min. The supernatant was removed and the residue in the tube served as the rhizosphere soil (R). The roots were then cut into segments of approximately 4 cm and stored in another centrifuge tube. A PBS-S solution [15] (phosphate buffer: NaCl: 17.60 g/L, Na_2_HPO_4_: 2.51 g/L, NaH_2_PO_4_: 0.47 g/L, and Silwet-77: 0.2 mL/L) was added to each tube and vortexed for 5 min. The roots were removed, and the tube was centrifugated at 16,000× *g* for 4 min. The supernatant was removed and the residue in the tube was collected for microbial DNA extraction of the external layer (E) of the iron plaque. The vortexed roots were placed in a new tube into which 0.5 M hydroxylamine-0.5 M hydrogen chloride solution and 3 mm diameter sterile glass beads were added. Hydrogen chloride solution can fully destroy the iron plaque structure and release the microorganisms [6]. Afterwards, the centrifuge tube was manually shaken for 5 min until the root surfaces became white and clean. The solution was collected into a new tube and centrifugated at 16,000× *g* for 4 min. The supernatant was removed and the residue in the tube was used for microbial DNA extraction of the internal layer (I) of the iron plaque. All the samples were stored at −20 °C prior to DNA extraction.

### 2.4. DNA Extraction

The DNA of bacteria in the non-rhizosphere soil (NR), rhizosphere soil (R), and internal (I) and external (E) layers of iron plaque samples was acquired by the Fast DNA SPIN Kit for Soil (MP Biomedicals, Illkirch, France). The extraction procedures were conducted according to the kit instructions. The DNA was extracted from the same amount of sample (500 mg) on the day after sampling. The extracted DNA was dissolved in 50 μL TE (10 mM Tris-HCl, 1 mM EDTA, pH 8.0) buffer and stored at 4 °C until further use.

### 2.5. Sequencing and Data Analysis

16S rRNA gene sequencing for the V3–V4 region of all extracted DNA was conducted by Shanghai Majorbio Biomedical Technology Co., Ltd. (Shanghai, China). An analysis of the sequencing data was conducted using the Bioinformatics Analysis Cloud Platform (http://www.i-sanger.com/) provided by the same company. In the analysis, only the top 20 most abundant bacterial genera were considered. Based on existing community abundance data, we used rigorous statistical methods to test the significant differences among groups, including species differences in abundance among samples of bacterial community of different groups. Then, an analytical test was conducted to evaluate the significance of the observed differences. Phylogenetic Investigation of Communities by Reconstruction of Unobserved States (PICRUSt) software package was used to investigate the bacterial functionality profile according to Langille et al. [16].

### 2.6. Simpson Index

The diversity of a bacterial community can be interpreted by Simpson’s phenomenon, described by Simpson in 1951. It is now commonly known as Simpson’s Paradox [17]. The formula is defined as follows:

$$D_{simpson}=\frac{\sum_{i=1}^{S_{abs}}n_{i}\left(n_{i}-1\right)}{N\left(N-1\right)}\;$$

where $S_{abs}$ is the number of observed OTUs, $n_{i}$ is the number of gene sequences contained in the *i*-th OTU, *N* is the number of all the gene sequences. The higher the value of the Simpson’s Paradox, the lower the diversity of the bacterial community.

### 2.7. Statistical Methods

The detection limit within two standard deviations was considered to be zero. The experimental data were organized by Excel 2016 (Microsoft Inc., Redmond, WA, USA) and subjected to a one-way ANOVA and single-factor analysis of variance with the SPSS statistical software package ver. 16.0 (SPSS Inc., Chicago, IL, USA). Significant differences were evaluated at *p* < 0.05. Figures were produced using Origin 8.5 (OriginLab Inc., Northampton, MA, USA).

## 3. Results and Discussion

### 3.1. Separation of Iron Plaque on Roots of Typha latifolia

**Figure 1:**
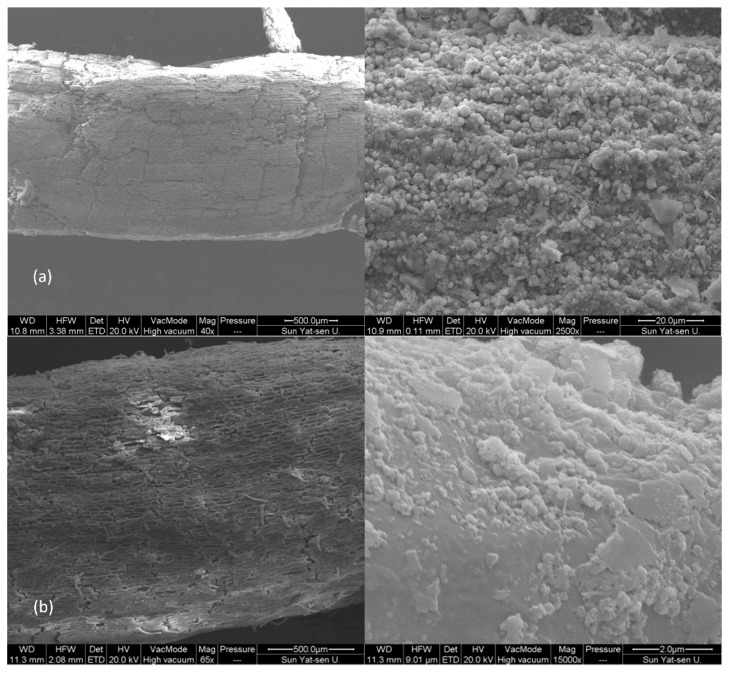

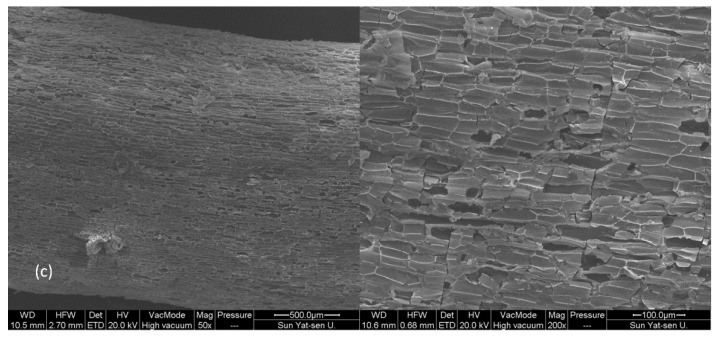
The SEM images of a *Typha latifolia* root segment from tailing soil treatment cleaned in steps by (**a**) deionized water; (**b**) PBS-S solution and (**c**) hydroxylammonium chloride solution. The images at the right show magnified regions of the images at the left. The round material above the root surface in (**a**) is external layer of iron plaque. The residues attached to the root surface in (**b**) are internal layer of iron plaque. The smooth, trellis-like structure in (**c**) is the root surface.

All plants survived to the end of the experiment. *Typha latifolia* planted in the non-polluted soil (NP) had dense foliage and strong stems and showed no signs of physiological stress. However, the growth of plants in the tailing soil (T) was negatively affected. Plant height in the T (approximately 40 cm in average) was 10 cm shorter than that in the NP. In addition, some areas of the leaves had become brown, and the stems were weak. However, iron plaque was found on the root surface as further evidenced by the SEM images in Figure 1a upon harvesting all the plants regardless of soil type (NP or T).

In this study, a successful separation of the external (E) and internal (I) layers of the iron plaque (IP) will guarantee the accurate interpretation of sequencing data concerning the bacteria in different IP layers. Though it has been well documented that the thickness of the IP can reach 100 µm [4,18], the IP cannot exactly be physically separated into external and internal layers for sample collection. Thus, the sequenced washing method based on chemical processes was developed in this study. The *Typha latifolia* root surfaces, either obtained from the NP or T treatment, were observed by SEM at each step of the sequenced washing to evaluate the separation effectiveness. As shown in Figure 1 with representative SEM images of a root segment from the T treatment, the IP remained firmly attached to the root surface after washing with deionized water (Figure 1a). Subsequent washing with PBS-S solution loosened the external layer of IP and resulted in its removal (Figure 1b). After the final washing with hydroxylamine hydrochloride solution, the IP was barely visible on the root surface (Figure 1c). Though the separation of the IP layers was not perfect from the perspective of exactly physical separation, marked differences were observed, as could be found in either Figure 1 or the upcoming analyses. Thus, a successful separation was achieved, guaranteeing the quality of collection and bacterial sequencing of the R, E and I samples.

### 3.2. Bacterial Community Distribution

**Figure 2:**
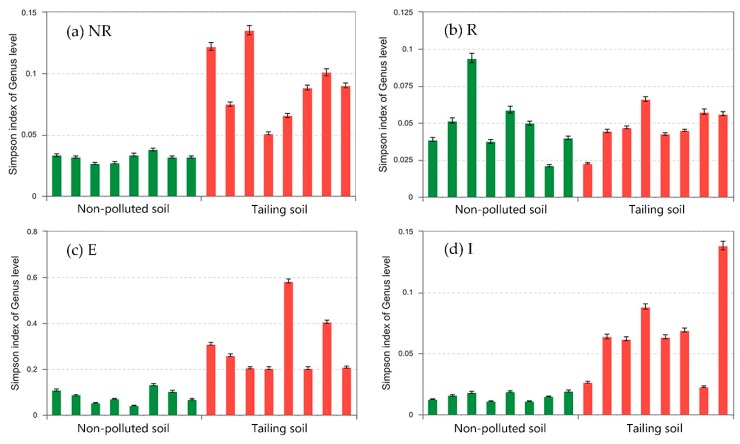
The bacterial community diversities in (**a**) the non-rhizosphere soil (NR); (**b**) the rhizosphere soil (R); (**c**) the external layer of the iron plaque (E) and (**d**) the internal layer of the iron plaque (I). The green bars are Simpson index values of 8 samples in the non-polluted soil (NP) while the red bars are those in the tailing soil (T). Error bars (SD, *n* = 8, *p* < 0.05) indicate the upper and lower bounds of the Simpson index in each sample.

Figure 2 shows that the diversities of the bacterial communities in the non-rhizosphere soil (NR), the external layer of iron plaque (E) and the internal layer of iron plaque (I) in the non-polluted soil (NP) were significantly higher than those of the corresponding zones in the tailing soil (T). In the NP, the alpha diversity index values of the NR, R, E, and I zones were 0.03, 0.05, 0.08 and 0.02, respectively. Thus, the diversities of the bacterial communities were in the following order: I > NR > R > E. It can be inferred that iron plaque separated the root surface from the soil, causing a significant difference in the distributions of bacteria between I and E. According to Flemming and Wingender [8], a large number of extracellular polymeric substances (EPS) were expected to distribute immediately at the root surface and hence can allow for much higher bacterial community diversity in the I zone. However, the iron plaque absorbed or coprecipitated with heavy metals (though presented in very low concentrations in the NP) to retain most of them in the E [19], preventing some sensitive bacteria from existing there. Similar phenomenon was also found in the T treatment. In the T, the alpha diversity index values of the NR, R, E, and I zones were 0.09, 0.05, 0.30 and 0.07, respectively. Thus, the order of bacterial community diversity for these four zones in the T was R > I > NR > E. By comparing the bacterial community diversity orders in both soil types, and regardless of the R zone in the T as well, it is reasonable to tell that the I zones in both soil harbor the most diverse bacterial communities. This might be attributed to, apart from the abovementioned EPS, the bacteria’s most direct access to the roots exudates since the I zones were closely attached to the root surface. As explained by Chabbi et al. who studied the wetland plant bulbous rush in acid mining lakes, the roots contained iron plaques surrounding a bacterial community at their surface, which produced a protective environment to allow for bacterial rapid recycling of carbon exuded by roots and hence obvious presence of microorganisms at the place approximately similar to the I zones in this study [20]. Therefore, an unknown regulating mechanism closely related to the soil physicochemical properties was believed to exist in the T and nevertheless required further study.

### 3.3. Predicted Functional Potential of Bacterial Communities

**Figure 3:**
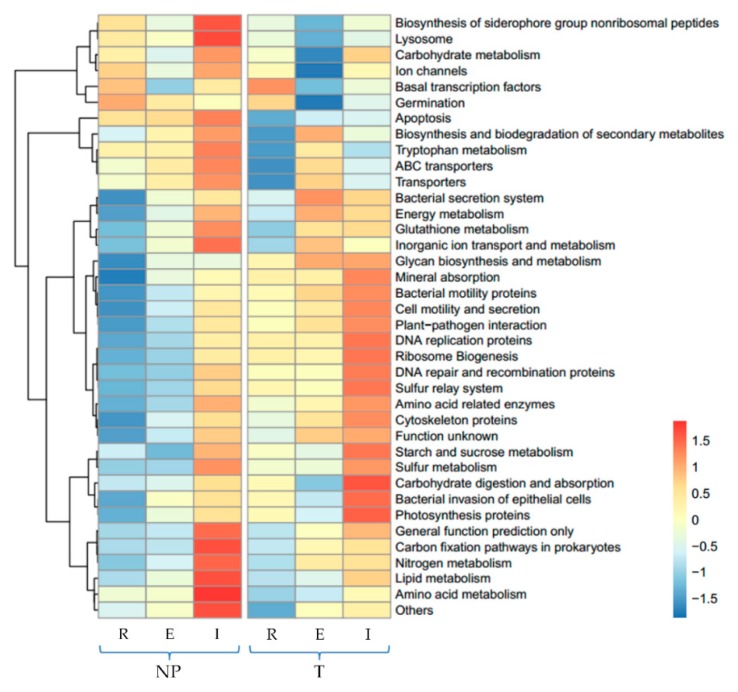
The heatmap of the relative abundance of some functional gene families of the internal layer (I) and external layer (E) of iron plaque and the rhizosphere (R) in non-polluted soil (NP) and tailing soil (T) predicted by PICRUSt.

PICRUSt is a bioinformatic tool that predicts metagenome gene functional content emerging in the recent 5 years [16]. In this study, we focused on the functional genes related to the metabolisms of iron and sulfur and circulation of C and N compounds. Among the 38 most abundant individual gene abundances predicted by PICRUSt in Figure 3, though no iron metabolism related gene was found in either soil type, siderophore biosynthesis related gene was indeed found with especially higher relative abundances in the NP. As shown in Table 1, the available iron was very low (0.63 mg/kg) in the NP, thus more siderophore was reasonably inferred to be synthesized by bacteria for acquiring adequate iron from the soil. Sulfur relay and metabolism related genes were higher in abundance in the I zone in the T, which could be explained by the very much high sulfur concentration in the T presented in Table 1. In terms of the C and N compounds circulation related genes, carbon fixation, nitrogen metabolism, lipid metabolism and amino acid metabolism genes were found most abundant in the I in the NP, while carbohydrate, starch, sucrose and amino acid circulation related genes were most abundant in the I in the T.

The PICRUSt heatmap (Figure 3) shows that the predicted functional genes content in the I was significantly higher than that in other zones in both NP and T. As described in Section 3.2, the I zone was found to exhibit high bacterial community diversity. The higher abundances of genes related to iron, sulfur, carbon, nitrogen metabolism/circulation in the I zone than in the other zones indicated that the I zone contributes most to the survival of *Typha latifolia*. According to Figure 3 and Table 1, it can be speculated that the higher abundances of functional genes in the I of either soil are attributed to the special environment of the root surface that was more affected by the root radial oxygen loss and the physicochemical properties of the soils. Similarly, Hou et at predicted the functional genes of the bacterial communities in the rhizosphere of *Sedum alfredii*, a hyperaccumulator, and proposed that the compositional difference of predicted functional genes was a consequence of the special environment of the rhizosphere, which was severely polluted by heavy metals in their case [21]. Although PICRUSt is useful for supplementing 16S rRNA analyses in metagenome studies, it might lead to confusion when analyzing microbial communities that have a large proportion of poorly characterized populations [21]. As a result, whole metagenome profiling and metatranscriptomic sequencing via sufficient geochemical analyses should be conducted to identify the connections between microbial taxonomy and biogeochemical functions.

### 3.4. Distributions of the Dominant Bacterial Phyla

**Figure 4:**
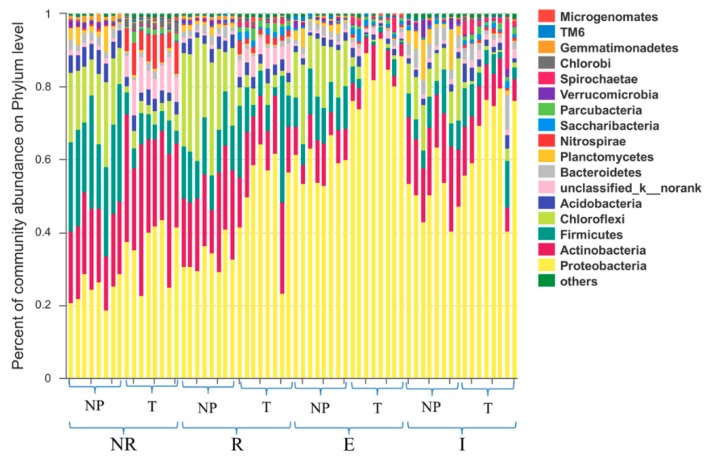
The distribution of the dominant bacteria at the phylum level in the non-rhizosphere (NR), rhizosphere (R), external layer of iron plaque (E) and internal layer of iron plaque (I) in the non-polluted soil (NP) and tailing soil (T).

The distributions of the dominant bacterial phyla in the NR, R, E and I zones of the non-polluted soil (NP) and tailing soil (T) are shown in Figure 4. Similar distribution patterns of the dominant bacterial phyla among the different zones were found. The four most abundant bacterial phyla in descending order of abundance were *Proteobacteria*, *Actinobacteria*, *Firmicutes,* and *Chloroflexi*. These four bacterial phyla account for approximately 80% of the total abundance. Figure 4 also shows that the dominant bacterial phyla differed among the different zones. Regardless of soil type, the abundance of *Proteobacteria* was much higher in the E zones than in the other zones. In addition, the abundance of *Actinobacteria* was higher in the NR zone than in the other three zones. In all four zones, the abundances of *Firmicutes* and *Chloroflexi* were much higher in the NP than in the T. The abovementioned four phyla were commonly found dominant in soil and this feature also remains in heavy metal polluted soils, though with variations depending on diverse environmental impact factors, as proven by recent studies on the root-associated microbiomes in polluted soils [22,23]. This current study showed that soil types and sampling zones, as the main drivers of environmental impact factors including heavy metal stress and oxygen supply, largely explained the microbiomes variation. Figure 4 also shows that *Acidobacteria* was present in all zones of the NP and T, and its abundance in the two soil types was comparable. These findings indicated that the four zones of the NP and T were acidic to some degree because *Acidobacteria* lives exclusively in acidic (low pH) environments [24,25]. As shown in Figure 4, *Nitrospirae* mainly existed in the T and was rare in the NP, which indicates a stress response of this nitrogen circulation related phylum to the lack of elemental nitrogen in the T as shown in Table 1. To cope with the insufficient nitrogen supply and hence potentially facilitate the plant growth in the T, the increased abundance of *Nitrospirae* was inferred to be compelled for possible nitrogen acquisition. Moreover, *Parcubacteria* was only found in the T. The bacteria of this phylum possess reduction functional genes [26] and participate in the nitrogen metabolism of plants [27]. *Gemmatimonadetes* was found only in the NP. Thus, the substantial variations in bacterial community were observed within and between the two different soil types (NP and T).

### 3.5. Dominant Genera of the Bacterial Communities

**Figure 5:**
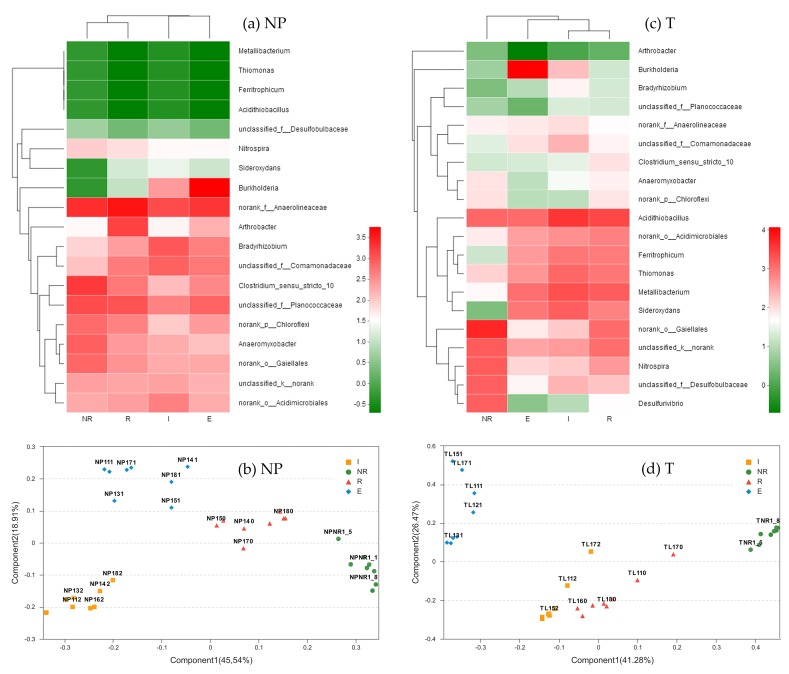
The heatmap analysis of bacteria at the genus level in the non-rhizosphere (NR), rhizosphere (R), external layer of iron plaque (E) and internal layer of iron plaque (I) in (**a**) the non-polluted soil (NP) and (**c**) the tailing soil (T). The heatmap plot depicts the relative percentage of each bacterial taxon (variables clustering on the Y-axis) within each sample (X-axis clustering). The relative values for the bacterial taxa are represented by color intensity according to the legend. The PCoA analyses of bacteria at the genus level in the NR, R, E and I zones in (**b**) the NP and (**d**) the T. The percentages of the diversity distributions explained by each axis is indicated on the figure. Samples associated with the I (yellow squares), NR (green circles), R (red triangles) and E (blue rhombuses) zones are shown as single points.

Figure 5a shows a community heatmap of the bacterial genera in the non-polluted soil (NP). Samples of the NR and R zones clustered together, and samples of the E and I zones also clustered together. The abundance of *Burkholderia* was much higher in the E zone than in the other three zones. Previously, *Burkholderia* has been extensively reported resistant to many heavy metals, especially Pb and Cd, and it can contribute to the growth and tolerance to heavy-metals of plants via its plant-growth promoting characteristics, which enhances phytoremediation [28,29]. Thus, the heavy metal tolerance of *Typha latifolia* might be intensified by *Burkholderia* in the external layer of its root iron plaque, which is a potential evidence of the plant’s survival strategy. Based on the PCoA method, a similar clustering pattern of the four zones was obtained, as shown in Figure 5b. Samples of the NR and R zones clustered together on the component 2 axis, and the samples of the E and I zones clustered together on the component 1 axis (Figure 5b).

A community heatmap of the bacterial genera in the tailing soil (T) is shown in Figure 5c. Samples of the R, E and I zones clustered together, distinct from those of the NR zones. Similar to the finding in the NP, in the T, the abundance of Burkholderia was higher in the E zone than in the other three zones, this result provides further evidence that the bacterial resistance to heavy metals mainly occurs in the E zone of the iron plaque, reducing heavy-metal stress to Typha latifolia. Due to the presence of iron and the high concentration of sulfur in the T (Table 1), the dominant Fe- and S-metabolizing bacteria belonging to the genera Acidithiobacillus (Fe and S oxidizer), Ferritrophicum (Fe oxidizer), Thiomonas (S oxidizer), Sideroxydans (Fe oxidizer) and Metallibacterium (Fe reducer) showed higher abundances in this soil than in the NP [30,31]. As shown in Figure 5c, Acidithiobacillus had absolute and relatively high abundance among the bacteria in all four zones. Acidithiobacillus is a genus of bacteria capable of Fe and S oxidation that can be favored in acidic environments [25]. This observation indicates that the four zones had higher levels of acidity in the T than in the NP, which is consistent with the lower pH value in the T (Table 1). Moreover, the abundance of Acidithiobacillus was the highest in the I zone; this finding was expected because the radial oxygen loss from the plant root produces an aerobic environment right at the rhizoplane where the I zone is attached. As in the heatmap analysis, the samples of R, E and I clustered together in PCoA analysis and formed clusters distinct from the NR samples on the component 1 axis, as shown in Figure 5d.

### 3.6. Genus Differences

#### 3.6.1. Difference in Species Abundance among the NR, R, E, and I

**Figure 6:**
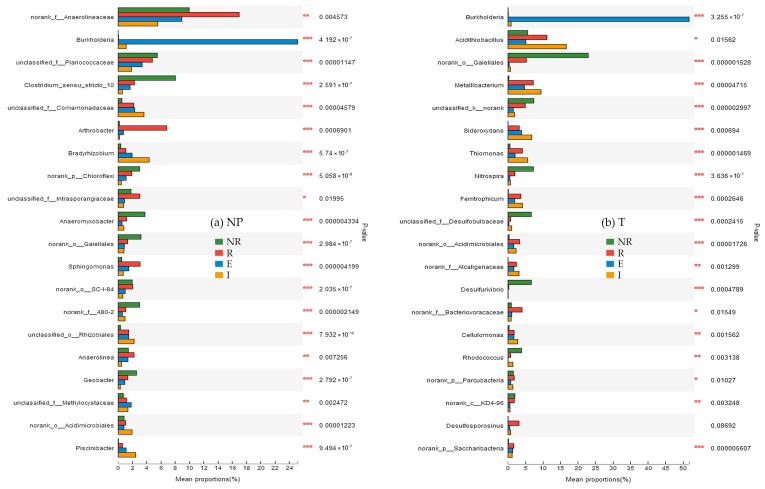
One-way ANOVA bar plots showing the differences in species abundance among the non-rhizosphere (NR), rhizosphere (R), external layer of iron plaque (E) and internal layer of iron plaque (I) in (**a**) the non-polluted soil (NP) and (**b**) the tailing soil (T). The ordinate shows taxon names of different levels, and the abscissa shows the abundance percentage of a taxon in the sample. (0.01 < *p* ≤ 0.05 *, 0.001 < *p* ≤ 0.01 **, *p* ≤ 0.001 ***).

As shown in Figure 6a, significant differences in species abundance among the four zones of non-polluted soil (NP) were identified though ANOVA at the genus level. Among the known bacterial genera, the largest significant difference in abundance was observed for *Piscinibacter*, whereas the smallest was observed for *Anaerolinea*. The *p*-value for *Piscinibacter* and *Anaerolinea* were 9.494 × 10^−7^ and 0.004256, respectively.

As shown in Figure 6b, similar to the corresponding analysis of the NP, the analysis of species abundance in the four zones of the tailing soil (T) via ANOVA at the genus level revealed significant differences among the four zones. *Thiomonas* exhibited the largest significant difference in abundance, whereas *Acidithiobacillus* exhibited the least. *Thiomonas* is commonly found in environments contaminated with arsenic [32], and it can oxidize thiosulfate, sulfite, S^0^ and hydrogen sulfide to sulfuric acid [33,34]. As shown in Figure 6b, the abundance of *Thiomonas* was the highest in zone I. This finding indicates that the oxidation of low valence-state sulfur to sulfate mainly occurs in the internal layer of the iron plaque, allowing plants to absorb and utilize sulfur. The abundance of *Ferritrophicum* and *Metallibacterium* in the four zones from highest to lowest was as follows: I > R > E > NR. *Ferritrophicum* is a type of iron-oxidizing bacterium and participates in the biogeochemical cycles of iron. However, its influence on ferric oxidation rates is unclear. It can be speculated that the oxidation of Fe^2+^ mainly occurred in the zone I of the T. *Metallibacterium* is a type of facultative anaerobe that can adapt to different acidic environments [35].

#### 3.6.2. Differences in Species Abundance between E and I

**Figure 7:**
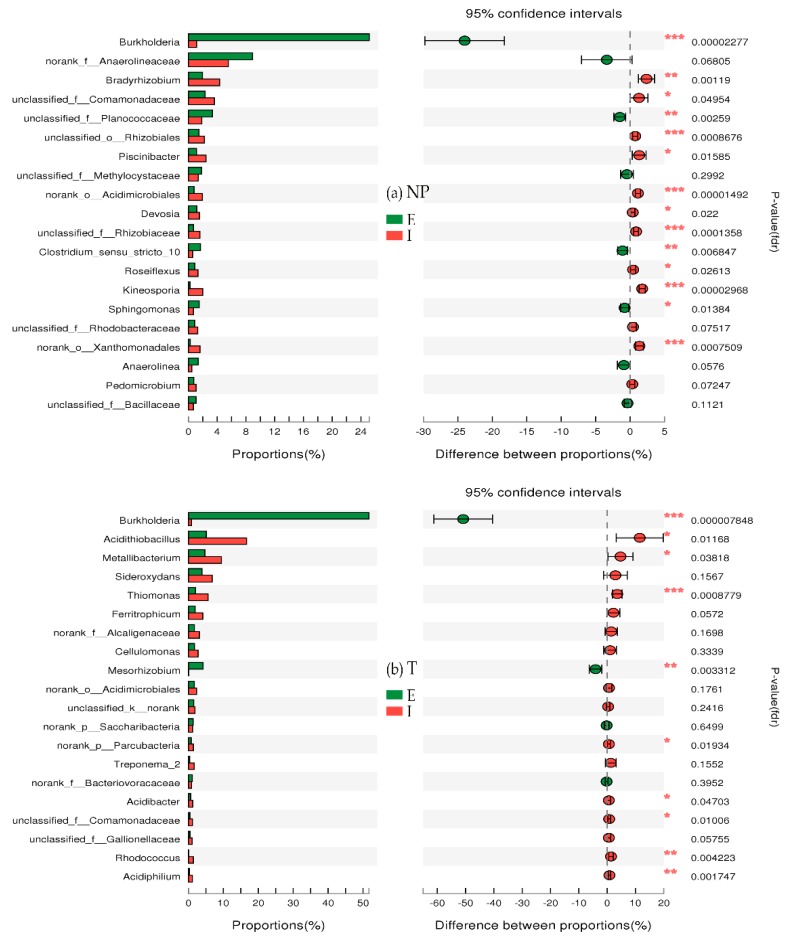
Differences in species abundances between the external layer (E) and internal layer (I) of iron plaque in (**a**) the non-polluted soil (NP) and (**b**) the tailing soil (T). (* 0.01 < *p* ≤ 0.05, ** 0.001 < *p* ≤ 0.01, *** *p* ≤ 0.001).

As shown in Figure 7a, based on a comparison of species abundances between E and I in non-polluted soil (NP), it was indicated that the bacterial communities of E and I were significantly different. Among the genera of known bacteria showing significantly higher abundance in the E than in the I, the order from highest to lowest was as follows: *Burkholderia, Clostridium_sensu_stricto_10*, and *Sphingomonas*. *Sphingomonas* is one of the main bacteria that can degrade aromatic compounds [36]. Moreover, it exhibits special activity as an antagonist against pathogenic fungi of plants [37]. Thus, the bacterial functions of resisting heavy metals and degrading aromatic compounds mainly occurred in E in the NP. Among the genera of known bacteria showing significantly higher abundance in the I than in the E, the order from highest to lowest was as follows: *Kineosporia Bradyrhizobium, Piscinibacter*, *Devosia*, and *Roseiflexus*. *Devosia* has nitration and nitrogen fixation abilities [38,39]. This observation indicates that microbial nitrogen fixation mainly occurs in I in the NP. Nitrogen fixing microbes can adapt to heavy metal polluted environments very well, and their abundances increase greatly in soil polluted with multiple metals and in other extreme environments that lack nitrogen [40,41]. *Roseiflexus* is a thermophilic filamentous photosynthetic bacterium that can grow in aerobic dark conditions by photosynthesis or heterotrophic growth [42]. Thus, it can exist widely in environments rich in nitrogen.

As in the non-polluted soil (NP), significant differences in species abundance between the E and I from the tailing soil (T) were found in Figure 7b. The abundances of *Burkholderia* and *Mesorhizobium* were much higher in the E than in the I. These intermediate rhizobia have nitration and nitrogen fixation abilities [43]. Bacterial resistance to heavy metals and nitrogen fixation mainly occurred in E in the T, which differs from the finding for the NP in Section 3.6.1. This result indicates that environmental stresses, i.e., low pH, high heavy metal concentrations in the T, can affect the nitrogen fixation of *Typha latifolia* and microorganisms on the root by changing the bacterial community structure. Among the genera of known bacteria showing significantly higher abundance in the E than in the I, the order from highest to lowest was as follows: *Thiomonas*, *Acidiphilium*, *Rhodococcus*, *Acidithiobacillus*, *Metallibacterium*, and *Acidobacteria*. The T used in this study was largely FeS_2_ based, such that the main reaction processes of FeS_2_ in the I zone of the T were the oxidation of FeS_2_ to Fe^2+^, SO_4_^2−^ and H^+^, and further oxidation of Fe^2+^ to Fe^3+^; these processes were influenced by microorganisms [44]. The abundance of *Thiomonas* was much higher in the I than in the E, which indicates that the oxidation of low valence state sulfur to sulfate mainly occurs in I. *Acidiphilium* is a genus of heterotrophic bacteria that exist in acid mineral environments [45], and can promote the reductive dissolution of ferric iron minerals indirectly [46]. These results suggest that the oxidation of ferrous ion and the reduction of ferric ions both occur in I in T.

It should be noted that, the iron and sulfur related processes mentioned above rarely occurred in the I zone of the NP, with nitrogen fixation by bacteria being the main process in the I (Figure 7a). As shown in Figure 7, significant differences in species abundance were found in both NP and T. In addition, the bacterial genera with significant differences in abundance between E and I were quite dissimilar between the NP and T. This finding shows that the differences in the physical and chemical properties between the NP and T in the current study led to differences in bacterial community structures.

## 4. Conclusions

The variations of the bacterial communities in the non-rhizosphere (NR), rhizosphere (R), external layer of iron plaque (E) and internal layer of iron plaque (I) of the wetland plant *Typha latifolia* were studied in a pot experiment for better understanding the survival strategy of wetland plant colonizing in extreme environment, e.g., mine tailing ponds rich in heavy metals. Based on Simpson index of genus level, the diversities of bacterial communities were found as I > NR > R > E in the non-polluted soil (NP) and as R > I > NR > E in the tailing soil (T). Higher diversities of bacterial communities were found in the NP, which was a result of the high C and N concentrations and low heavy metal concentrations therein. Regardless of soil type, the I zone harbored the most abundant functional genes, with those related to sulfur relay and metabolism more found in the T, and those related to iron acquisition and carbon and nitrogen circulation more found in the NP. Bacterial community structures were very different between the E and I zones in the NP and T. Many types of bacterial genera, including *Acidithiobacillus*, *Ferritrophicum*, *Thiomonas*, *Metallibacterium* and *Sideroxydans*, related to iron-sulfur cycling were found in the I zone in the T, suggesting much intense Fe- and S-metabolizing activities occurring there. Therefore, the I zone was the place to facilitate more bacterial processes related to iron and sulfur cycles. According to the analysis of dominant bacterial communities at genus level, the heavy metal resistant genus *Burkholderia* showed highest abundance in the E zones in both soils, which indicated bacterial antagonism to heavy metals was an important biological process in the external layer of the iron plaque.

The separation of the iron plaque into E and I zones by using a sequenced washing method was the first endeavor in the scientific world so far. Though the method was proven effective by SEM, a more precise separation of the E and I zones of iron plaque while avoiding breaking samples and an in-depth exploration of the biogeochemical cycles that occur in these zones are important topics to study in the future.

## Figures and Tables

**Table 1:** The physicochemical properties of the soils.

Physicochemical Property		Soil	
		T (Tailing Soil)	NP (Non-Polluted Soil)
pH		3.16 ± 0.02	5.96 ± 0.06
Total (mg/kg)	C	500.00 ± 100.00	12,766.67 ± 650.64
	N	n.d.	1066.67 ± 57.74
	S	101,200.00 ± 2179.45	n.d.
Total (mg/kg)	Pb	78.48 ± 5.91	40.75 ± 2.13
	Zn	492.05 ± 31.88	76.01 ± 6.02
	Cu	3048.95 ± 153.24	32.40 ± 0.76
	Cd	3.26 ± 0.37	0.37 ± 0.09
	Fe	15,851.22 ± 2443.78	21,094.82 ± 280.14
NH_4_NO_3_-extractable (mg/kg)	Pb	2.26 ± 0.42	0.02 ± 0.02
	Zn	96.23 ± 4.06	0.27 ± 0.13
	Cu	99.67 ± 3.89	0.29 ± 1.60
	Cd	0.33 ± 0.02	0.03 ± 0.00
	Fe	835.69 ± 17.79	0.63 ± 0.26
